# A tunnel coaxial 3D hyperspectral scanning system for underground mine investigation

**DOI:** 10.1038/s41598-023-37565-4

**Published:** 2023-06-27

**Authors:** Hyunseob Baik, Young-Sun Son, Kwang-Eun Kim

**Affiliations:** 1 Department of Resources Engineering, KIGAM School, University of Science and Technology (UST), Daejeon, 34132 Republic of Korea; 2 Mineral Resources Research Division, Korea Institute of Geoscience and Mineral Resources (KIGAM), Daejeon, 34132 Republic of Korea

**Keywords:** Civil engineering, Imaging and sensing

## Abstract

A hyperspectral scanning system was developed for three-dimensional (3D) surface mapping in underground spaces, such as mine shafts and tunnels. A hyperspectral line-scanning camera was mounted on the rotating driver unit coaxial with the tunnel to image both the mine wall and the ceiling. Uniform light was illuminated on the target surface to be imaged using a halogen lamp rotating together with the hyperspectral imaging sensor. Inertial Measuring Unit (IMU) was also attached to the sensor unit together with the hyperspectral camera so that sensor’s geometric information could be acquired simultaneously during imaging. All sensor and controller units were mounted on a cart-type platform for easy movement in the tunnel, and a battery mounted on the platform supplied power for system operation and the halogen light source. The developed scanning system was tested in an actual mine, and 3D hyperspectral images of the internal surface of the mine shaft were successfully obtained.

## Introduction

Mines are typical harsh workplaces with high demand for unmanned and automated operations. Thus, major mining companies in the United States of America (USA) and Australia are developing and applying smart mining technologies, including unmanned mines^1–7^. Smart mining enables optimal and efficient mining and very high level of safety by minimizing manpower input for mining, transportation, drilling and blasting through state of art sensing technologies and unmanned automation technology. For automated smart mining, it is necessary to determine the value of rocks as minerals at the mine end site and also to figure out the spatial distribution of useful minerals in near real time. In other words, for smart mining, it is necessary to obtain information about the geological structure and mineral composition of the mining end in near real time. However, until now, the collection of information on the mineral composition and distribution at a mine site relies almost entirely in site visual inspection by geologists and geochemical analysis of some rock samples^8^.

Recently, hyperspectral remote sensing technology has attracted considerable attention as a technology that can replace the fieldwork of geological experts^9^. The ability of hyperspectral remote sensing technology to detect and discriminate geologic materials has the advantages of expert level in terms of accuracy and speed, and expanding and developing an element sensing technology for smart mining is easy.

Hyperspectral imaging is a technology that acquires tens to hundreds of continuous spectral information of materials located in each pixel of an image by spectroscopy of light incident on a sensor^10–12^. From hyperspectral images (HSI), unique optical properties exist for each material, and the absorption and reflection characteristics can be analyzed. Owing to these advantages, hyperspectral imaging has recently been adopted in the mining industry^13,14^. In particular, drone-based hyperspectral remote sensing technology has been confirmed as a highly useful tool in open-pit mining sites for mineral detection and monitoring^15^.

Various studies have attempted to utilize hyperspectral technology not only in the air but also on the ground and underground. Hyperspectral sensors mounted on ground-based platforms have been used for the geological analysis of outcrops on the surface^14,16–20^. Light Detection and Ranging (LIDAR) and stereo photography are also used very often in underground mining for the mapping of subsurface geology and geologic structure^21–23^. Recently, multispectral Light Detection and Ranging (LIDAR), which analyzes the spectrum of returned light, was developed and successfully applied for the discrimination of ores and gangue in mine shafts^24,25^. Turner et al.^26^ developed an unmanned drone system that mounted a multispectral camera, thermal imaging camera, and LIDAR to map geological discontinuities during excavation in an underground mine. However, many technical problems remain in using unmanned drones in underground environments, because GNSS (Global Navigation Satellite System) cannot be used in underground environments and Inertial Measuring Unit (IMU) often fails to function properly owing to the effects of steel structures or magnetic iron ore bodies.

This study proposes a ground-based active underground hyperspectral imaging system for the 3D surface geological mapping of underground mine shafts or tunnels. The proposed system can investigate subsurface geological structures and mineral deposits that are difficult to identify in conventional airborne or spaceborne-based hyperspectral remote sensing. The proposed underground hyperspectral exploration system is a quasi-quantitative exploration technique that can perform mineral distribution mapping by utilizing the quantitative signal strength and spectral characteristics of each target mineral. In particular, the results of this study can create a 3D model of ore body distribution.

**Figure 1:**
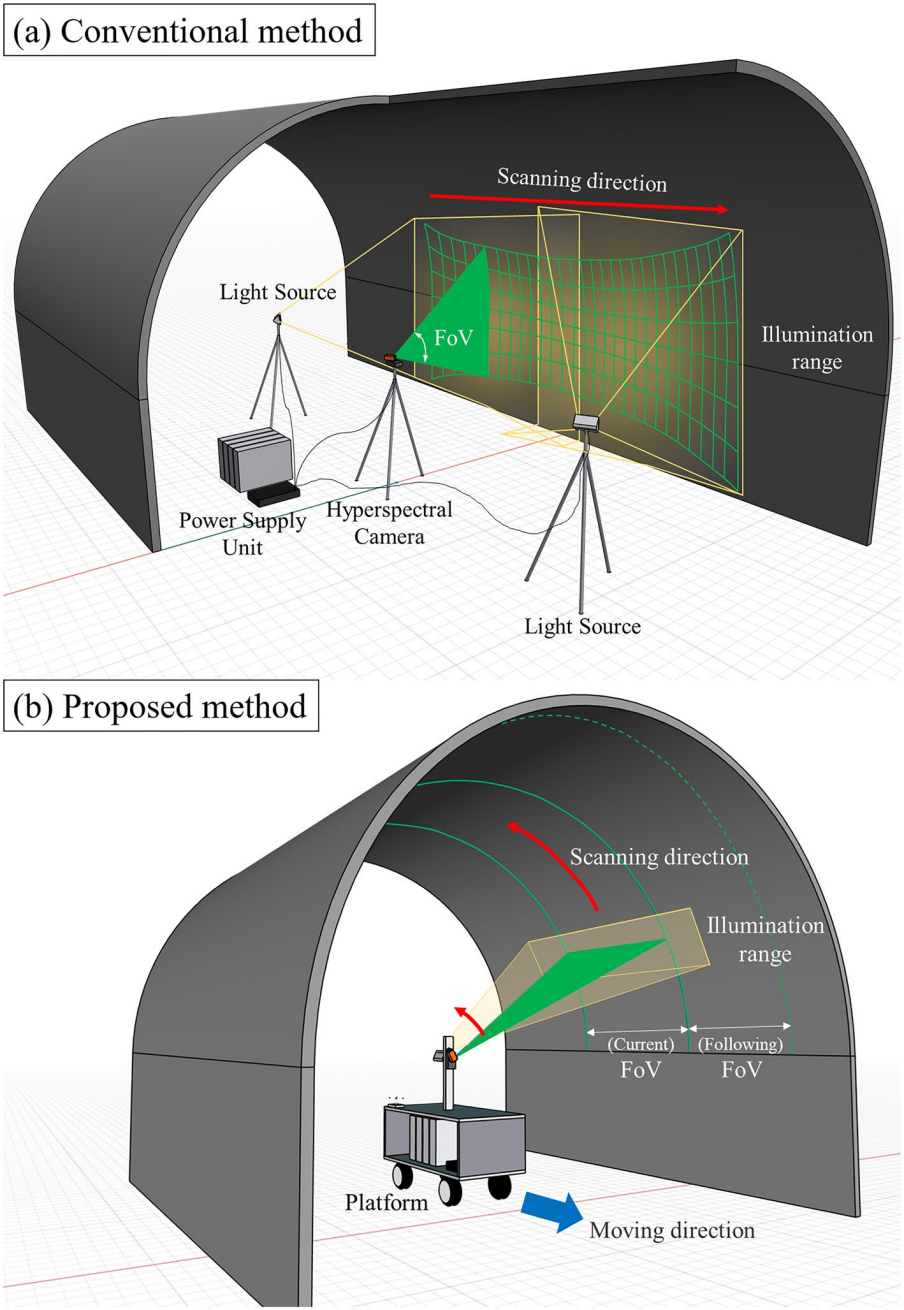
A schematic diagram of hyperspectral scanning system in mine shafts and tunnels. Pushbroom hyperspectral camera scans with the certain Field-of-View (FoV). (**a**) Conventional method^28^. The light sources are fixed; the boundary of the illumination range has lower irradiance than the center. Due to the horizontal rotation of hyperspectral camera, the image’s resolution varies by the viewing angle. The power supply unit is separate from the sensor system and is reinstalled with every movement of the exploration point. (**b**) Proposed method in this study. The hyperspectral camera and light source rotate coaxially with respect to the tunnel. The platform is stationary while one image strip is scanned and then moves to the following strip position when the scan is complete. The irradiance and resolution of the tunnel surface are kept homogeneous during the scanning. Power Supply Unit and cables are installed inside the platform for easy movement.

Most hyperspectral imaging sensors are designed using pushbroom cameras. Pushbroom imaging is a method of moving and scanning charge-coupled devices (CCDs) arranged in one line at a constant speed, and each line acquired from the CCDs is continuously arranged at a certain period (frame/second) to form an image^27^. In previous ground-based hyperspectral remote sensing studies, a pushbroom hyperspectral camera was mounted on a rotation stage installed on a tripod at a fixed point and set to rotate about an axis orthogonal to the ground surface^28^ as shown in Fig. 1a. The biggest problem in applying this conventional scanning method in underground tunnels is that it is impossible to acquire 3D images of the entire surface of the tunnels. Also, as this method uses a fixed field-of-view (FoV) of the sensor, the resolution and actual scanning range change depending on the distance between the target surface and the scanning origin. As shown in Fig. 1a, the resolution is high in the center of the image, while the scan range is narrow, and both ends of the image have low resolution and a wide scan range. The pushbroom scan of the outcrop plane at such a fixed origin generates a geometrically distorted image. In addition, since the light source is also installed at a fixed position, the intensity of the incident light source varies greatly depending on the position. Even if multiple light sources are installed, it is almost impossible to irradiate light on all target surfaces uniformly. For spectral analysis, the radiometric calibration process is crucial for obtaining accurate spectral reflection characteristics of the target material^29–31^. Therefore, a light source capable of uniformly irradiating all surfaces is required.

The proposed method (Fig. 1b) can improve the hyperspectral remote sensing for underground mines. The hyperspectral camera and the light source are mounted on a device rotating along the tunnel wall. Assuming that the surface of the mine tunnel is roughly half-cylindrical, the central axis of the motor coincides with the central axis of the half-cylinder. In this condition, the scanning range (FOV) of the camera and the light projected from the light source sweeps perpendicular to the surface of the tunnel. This method can dramatically reduce the distance deviation from the origin of the sensor to the tunnel’s surface compared to the conventional method, even considering the undulations of the tunnel. Also, the ceiling of the tunnel can be scanned by coaxial scanning. Compared to the walls of the tunnel, the ceiling surface is less accessible and difficult for mine workers to analyze at close range. Therefore, the proposed method to analyze the ceiling is more effective in mine outcrop survey than the conventional method. In summary, the proposed method enables the acquisition of images with uniform spatial resolution and light source effect over the entire tunnel surface.

Optical remote sensing detects the light reflected from the surface of a target object^32^. In traditional visible-infrared optical remote sensing research, natural light (sunlight) is used as a light source. As the target is the ground surface where sunlight is incident, most have used passive systems that do not have separate light sources. In hyperspectral remote sensing, reflectance spectrum is the key parameter which is analyzed for the detection of target material. In order to convert the measured brightness value into reflectance, information about the spectrum of the light source is essential. The hyperspectral remote sensing system in the air or on the ground is a passive system and it is easy to convert brightness into reflectance with the information on the spectrum of sunlight and the atmospheric effect.

However, most hyperspectral remote sensing system in indoor or underground uses a halogen lamp^33^ installed in a fixed position as a light source. When such a fixed lamp is used as an artificial light source and the surface to be imaged is large, it is very difficult to maintain a uniform illuminance over the entire target surface due to the large difference in distance to the light source for each target location and the directional characteristics of the halogen lamp. Using multiple light sources as shown in Fig. 1a can be an alternative but the repeating process of the lamp installation considering the condition for uniform illuminance can be very inefficient and time consuming.

In this study, we propose an active system in which the scanning direction of the hyperspectral camera and the illumination direction of the light source are synchronized (Fig. 1b). The most important advantage of the proposed method is that since the light source rotates with the hyperspectral camera, light can always be irradiated with almost uniform brightness in a direction perpendicular to the scanning plane, minimizing radiative distortion depending on the angle and intensity of the light source. Also, in the process of exploring a long tunnel, this system acquires hyperspectral images by moving the entire platform, which includes an image sensor, a rotating light source, and a power source. Therefore, the process of repeatedly installing and removing the light source and imaging sensor as in the conventional method is unnecessary, which can greatly improve the efficiency and speed of navigation as well as accuracy.

## Results and discussion

### Test site exploration

**Figure 2:**
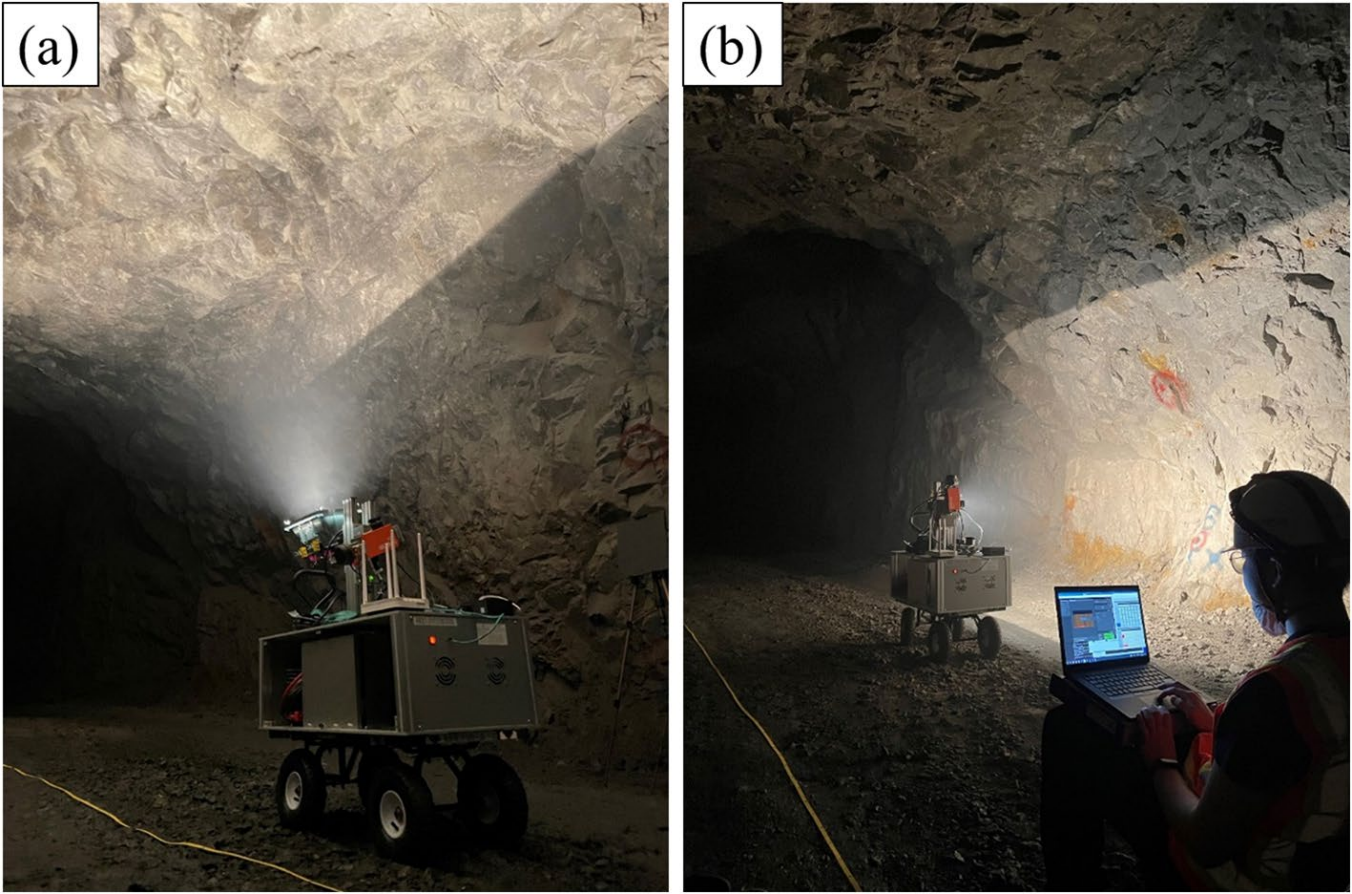
Test exploration at Gwan-In Magnetite Mine, Pocheon, South Korea. (**a**) Scan of the shaft ceiling during actual system operation. (**b**) Wireless control.

The proposed 3D hyperspectral scanning system was tested in the underground tunnel of the Gwan-In Magnetite Mine in Pocheon, Gyeonggi-do, Korea (Fig. 2). This mine produces high-grade magnetite and ilmenite through Fe–Ti–V mineralization in various ultramafic igneous rocks, such as gabbro, through magma differentiation in igneous rocks intruded in the Neoproterozoic (873–852 Ma). A rock phase in which gabbroic pegmatite intrudes into diorite and gabbro because of late igneous activity, and pegmatite is subdivided according to mineral composition such as pyroxene, olivine, plagioclase, and amphibole^34^. The Fe–Ti–V ore body in this area was investigated and developed in the early twentieth century, and mining remains in progress.

On August 18, 2021, a test exploration was conducted using the prototype equipment. The exploration site is a section of a mine tunnel located approximately 300 m underground (65 m above sea level), with an average tunnel width of approximately 5 m. Data acquisition was conducted for one station of a target section tunnel. A portable laser distance meter was used to locate the platform at the center of the tunnel. The main ore bodies in the shaft have already been mined, but some high-grade ores remain on the walls of the mines; therefore, iron ore and gangue alternately appear along the shaft. As the shaft is the northernmost part of the mine’s iron orebody, iron orebodies are distributed on the southern wall. Additionally, some fractures and joints derived from the fault movement process in this area were observed in the mine.

### 3D projected HSI and target detection

**Figure 3:**
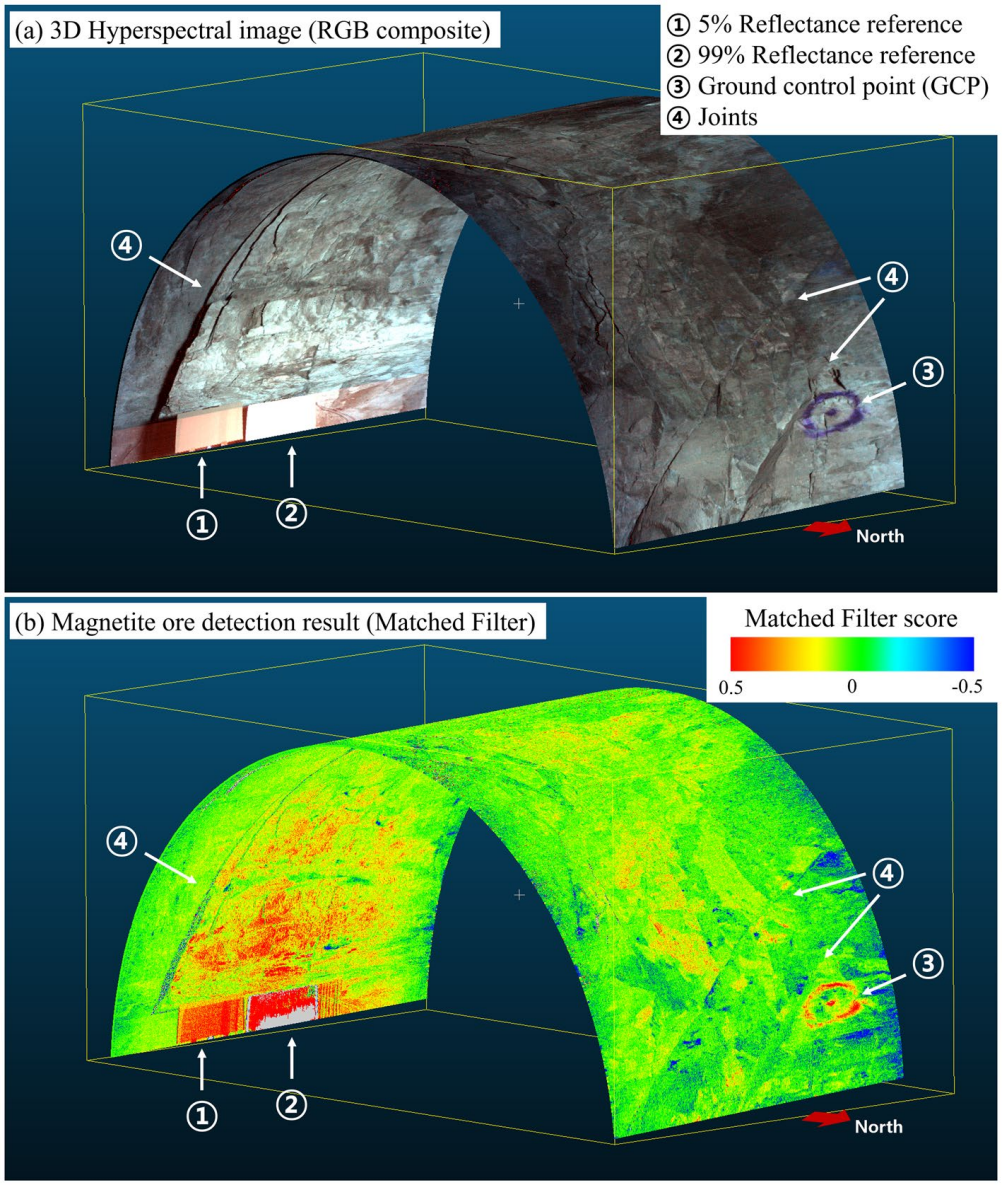
Result of hyperspectral 3D modeling, view from the northeast. (**a**) RGB composite. Point 1 and 2 is spectral reference panels of reflectance = 5% and 99%. Point 3 is ground control point (GCP) is marked by spray paint that has measured coordinates by triangulation in the mine. Points 4 are joints, which are thought to have been geologically formed by the force of the formation of fault. (**b**) Iron ore detection results. Gray was excluded as an abnormal value. Southern wall of the mine shows high matched filter values than the northern wall. The abnormal high detection results are shown in reference panels (Points 1 and 2) and GCP (Point 3).

Figures 3 shows the 3D projection and mineral mapping results of dataset acquired from test exploration. RGB synthesized hyperspectral 3D model is shown in Fig. 3a. Red, green, and blue pixels are 630, 532, and 465 nm wavelength bands. A half-cylindrical mine tunnel wall and ceiling were visualized based on the position information. Mine walls had a dark gray appearance. This surface color is characteristic of dark rocks, such as iron ore and gabbro. Two reflectance references (points 1 and 2) were observed on the southern mine wall, and GCP (point 3) was observed on the northern mine wall. A joint (point 4) appeared to the left of the reference panel and was distributed from the southern mine wall to the mine ceiling. Joints with slopes in the east direction developed on both the north and south sides. Regarding the line near the bottom of the south side, which includes the reflectance reference, pixels other than the reference are distorted in pixel value owing to the scattering effect in the air, showing a different color from the other shaft walls.

Figure 3b shows the result of mapping the distribution of iron orebodies using the matched filter algorithm for the hyperspectral 3D model. The matched filter is a technique that uses covariance to check the similarity between the spectral signal of a pixel and the target spectral signal and has been used in many hyperspectral geological mapping studies^11,35–37^. In this study, the target spectrum is the average value of 10 spectral libraries previously acquired with Specim® FX10 hyperspectral images from high-grade iron ore drilling core samples. As a result, a signal similar to a significant iron ore with a Matched filter score of 0.5 or higher was detected in the southern mine wall. In contrast, the iron ore signal was relatively low in the northern mine wall. The deflection of these iron ore signals is believed to be due to the distribution of iron ore, mainly in the south, as the mine shaft of this exploration corresponds to the outermost part of the main ore body.

**Figure 4:**
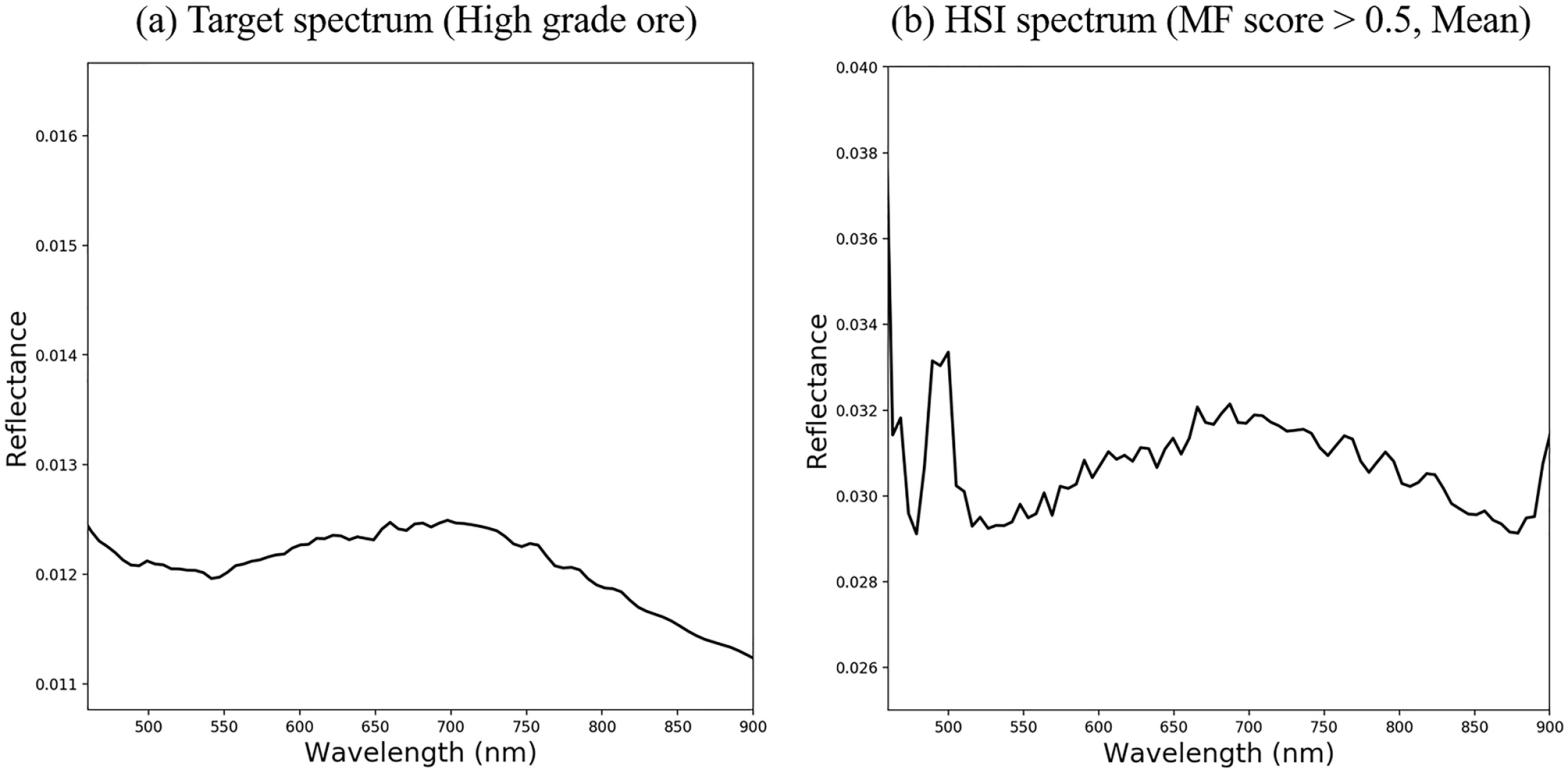
Comparison of the spectra of the target reference sample and the iron ore spectra obtained in the developed system. (**a**) Average values of spectral libraries from high-grade iron ore. It was used as the target spectrum of the Matched Filter algorithm. (**b**) The average spectrum extracted from the Matching Filter scored 0.5 or higher pixels among hyperspectral images acquired in the mine. Only 450–900 nm wavelength bands were compared, and pixels corresponding to the reflectance reference panel and GCP were excluded.

Although it does not correspond to iron ore, the matched filter score value was also high in the reflectance reference panel (points 1 and 2) and GCP (point 3). This is because of the spectral characteristics of iron ore. Magnetite and Ilmenite have weak peaks, such as absorption lines, compared to other minerals in the VNIR wavelength range, and the absolute value of reflectance is also low^38^. The reference panels provide a homogeneous 5% or 99% reflectance across all spectra, which is considered highly similar to iron ore’s weak spectral properties. As the GCP is marked with spray paint on the surface of the mine wall, it seems that the spectral signal from the paint was mistakenly detected as a signal similar to that of the iron ore. Figure 4b shows the average spectral curve of the hyperspectral image for pixels with a matched filter score of 0.5, excluding false detection pixels. Compared with the target spectrum (Fig. 4a) in the 450–900 nm wavelength band, the shallow rise of the spectral curve around the 700 nm wavelength band is consistent, and the Pearson correlation coefficient^39^ is 0.637, which is believed to have detected iron ore bodies.

The results of this study are considered to have influenced the accuracy by the following factors:

1) Mining dust and groundwater vapor: These factors can affect the radiometric calibration quality of hyperspectral image. The illumination and reflected light can be absorbed by dust and vapor. The radiometric calibration was performed using reference panels that relatively considered these factors, but it must be evaluated for future precise studies.

2) Vibration from mining activities: Posture information was measured in real-time from the IMU, during hyperspectral data is in acquisition. However, fine ground vibrations caused by mining activities (drilling, vehicle movement) have frequencies below the IMU’s data measurement frequency, which can shake the image obtained in the middle of the IMU data acquisition cycle. In this study, this problem did not appear because the section without mining work was used as a testbed, but it is expected to be a problem in places where mining activities are actively carried out, such as working face at the blind end. Therefore, it is believed that these factors should be considered in future empirical studies.

**Figure 5:**
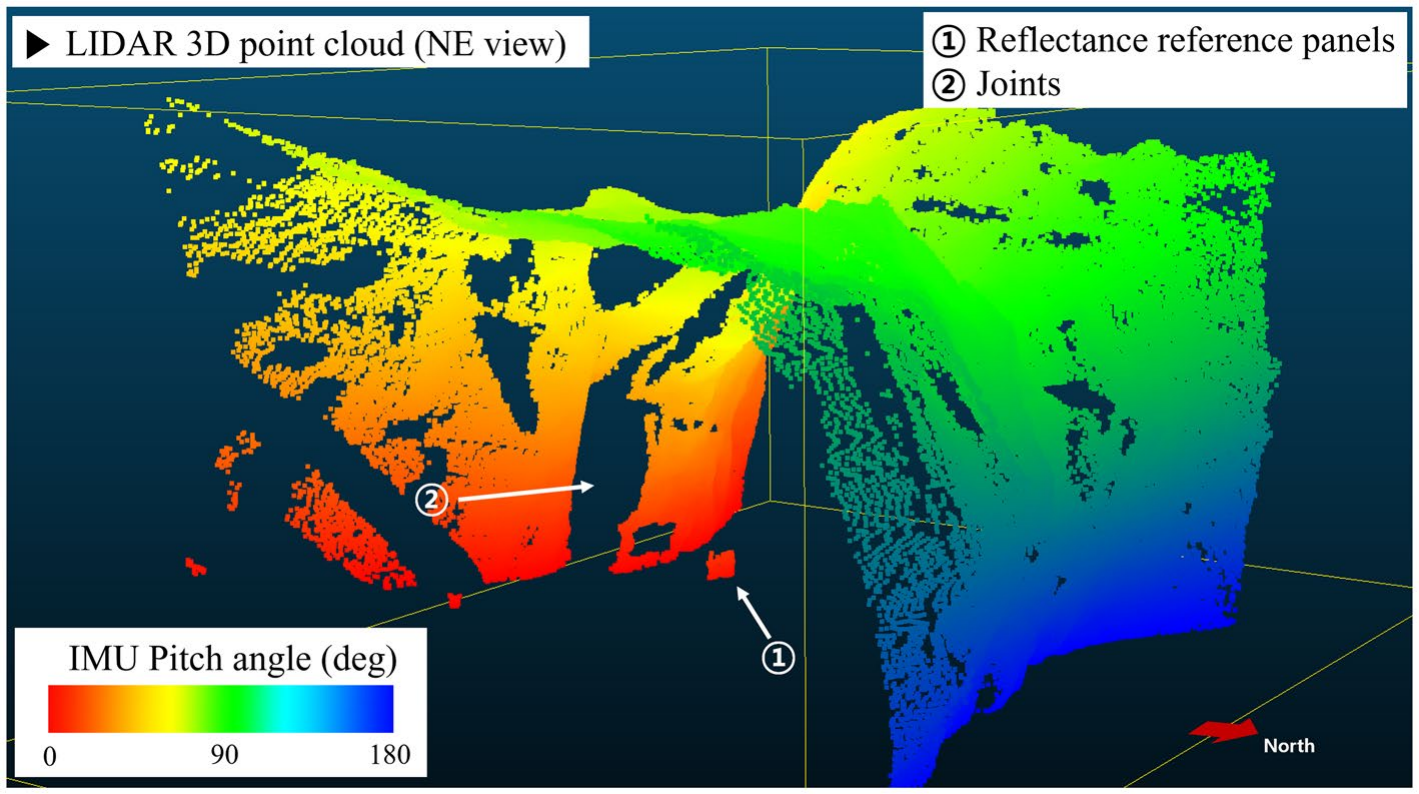
3D point cloud from LIDAR data. Auxiliary geometry data was used for setting the radius of the 3D hyperspectral model and for comparative visual analysis.

Figure 5 shows a projection of the auxiliary point cloud data from LIDAR. The curvature of the mine surface was modeled, and protruded parts (point 1) in Fig. 5 are the reflectance reference panels. Because of the support (tripod), the reference panel cannot be completely attached to the mine wall; therefore, the acquired geometric information appears to cover the mine wall and protrude. The LIDAR scan was performed from southern wall to northern wall. This data is used to determine the tunnel radius in 3D projection of hyperspectral data and visual comparison between the 3D models. Also, these data will be used in the precise 3D geometric projection of hyperspectral model in future works.

## Conclusion

In this study, a hyperspectral exploration system for underground spaces such as mines was designed and developed. The hyperspectral camera is configured to rotate coaxially with the shaft so that both the shaft wall and ceiling can be scanned. A directional halogen lamp light source was synchronized with the rotational speed of the camera to provide a homogeneous amount of light to the target surface, thereby establishing an active hyperspectral system. A stereo camera, LIDAR, and IMU were attached to produce a model that obtained geometric information and projected hyperspectral images in 3D space and was compared with 3D point clouds, including color information obtained from stereo cameras and LIDAR. This allowed direct comparison with the geological structure and shape of the mine. The system has built-in batteries, inverters, and wireless routers that enable wireless remote control and subsequent smart mining environments. All the equipment was assembled in a rubber-wheeled cart to increase mobility. As a result of acquiring pilot data on iron mines, it was possible to detect iron ore bodies and confirm their three-dimensional distribution.

Additional technology development is required to project the current hyperspectral model onto the real 3D shape of the mine shaft. We are currently developing a technology to implement a 3D hyperspectral model projected onto an accurate mine shaft model. Specifically, since each pixel of the hyperspectral 3D model has a 3-axis rotation angle with respect to the shooting origin, the pixel is applied to the auxiliary point cloud model at a point extending from the origin to the corresponding angle. When the development of the technology to project hyperspectral image pixels using IMU data onto the 3D geometry model created by LIDAR is completed, considering the precision and error range of IMU, it will be possible to create a very precise 3D subsurface geological map which sufficiently satisfy the accuracy and precision condition required in mining industry. Implementing an accurate model for each strip allows mosaicking of multiple strips with matching the GCPs. Through this, it will be possible to create a real 3D hyperspectral image model covering the entire mine shaft by collecting several strips obtained while moving along the shaft.

This system is expected to effectively investigate geological information exposed to underground outcrops, such as mineral deposit evaluation and 3D geological modeling. In addition, because of its excellent field applicability and mobility, periodic scanning is expected to help quickly detect and prepare for changes in vulnerable areas (e.g. faults, joints) inside the mine tunnel. Furthermore, it is expected to be used as a core sensing element technology for unmanned smart mining.

## Method

### System design

**Figure 6:**
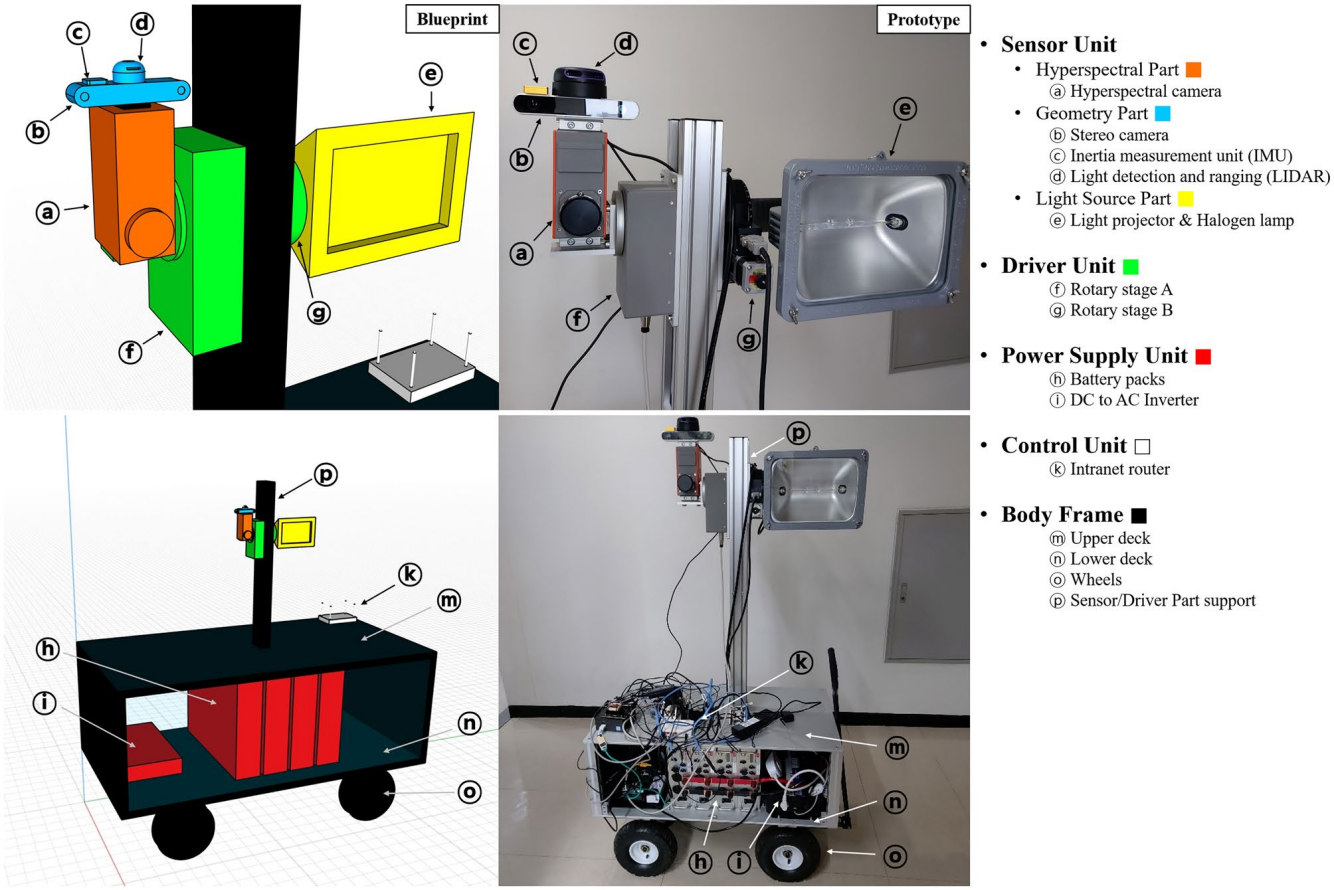
Blueprint and prototype image of the entire system. The Sensor Unit and the Driver Unit is displayed in the upper row by enlarging the blueprint and photograph in detail. The Hyperspectral Part and Light Source Part is fixed to the Driver Unit’s rotation axis. Rotary stages A and B rotate synchronously with each other.

**Figure 7:**
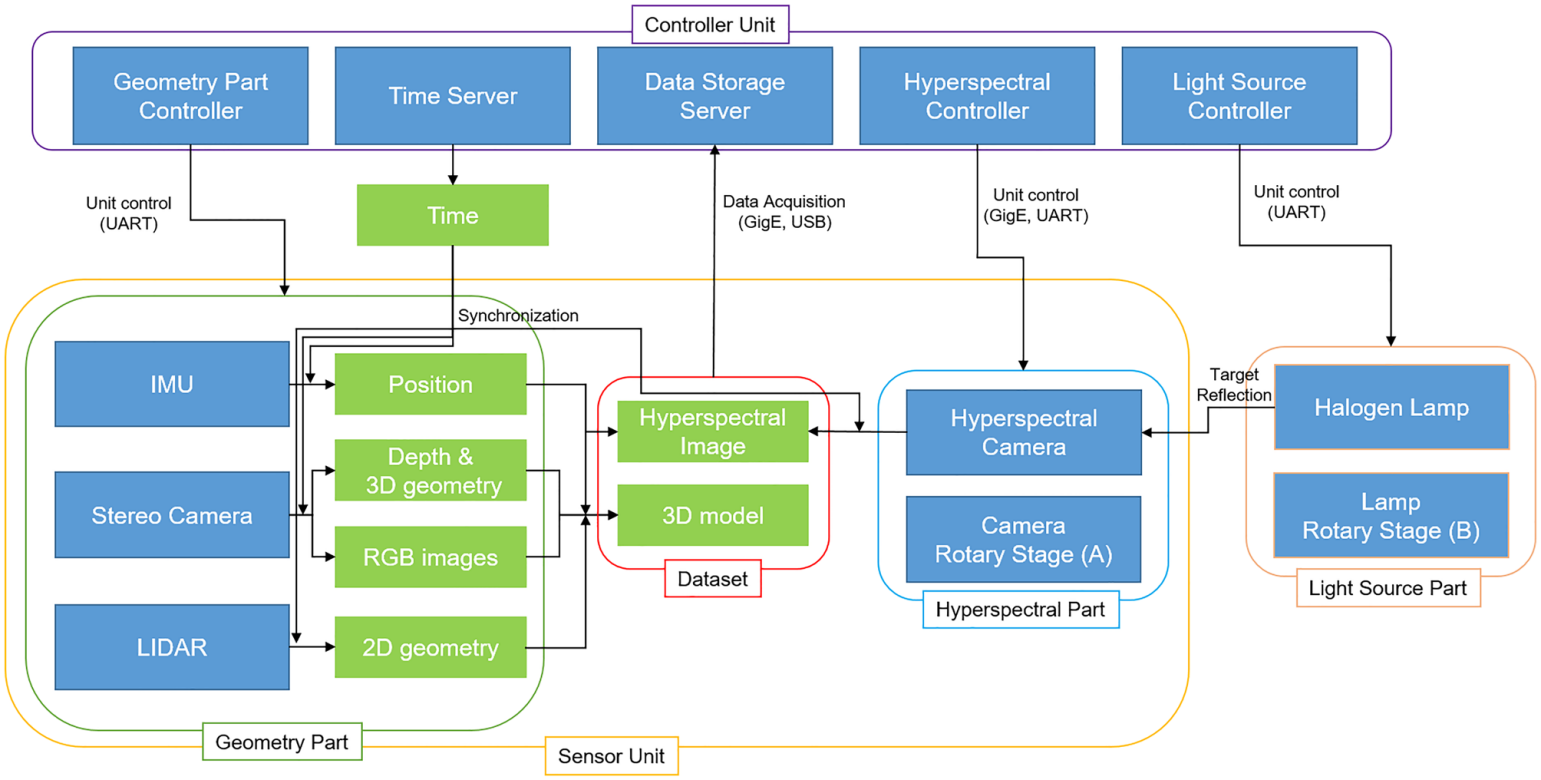
Schematic diagram. Blue box: hardware or software. Green box: output data. Geometry Part and Hyperspectral Part have synchronized time information via Time Server in Controller Unit for data synchronization. Each controller part controls data acquisition options. Hyperspectral Controller and Light Source Controller control the Rotary stages’ rotation velocity and angular position.

The Sensor unit includes a hyperspectral part, light source part, and geometry part (Figs. 6 and 7). The hyperspectral part is the core part for acquiring a hyperspectral image, and the light source part provides the light required for an active system. The geometric part acquires various geometric and positional data to assist with the hyperspectral image. The FX10 and FX17, which are pushbroom hyperspectral sensors from Specim, Finland, were mounted as cameras. FX10 has 448 bands in the 400–1000 nm visible-to-near-infrared (VNIR) wavelength range. One line is arranged in 1024-pixel CCDs. FX17 has 224 bands in the 900–1700 nm NIR wavelength range. One line is arranged in 640 pixels. Both cameras had an FOV of 38°. In one session of investigation, only one type of camera is selected. The GigE Vision protocol was used for the data acquisition and control. Raw hyperspectral images were stored as 12-bit DN (Digital Number) values between 0 and 4095, including L3Harris Geospatial ENVI® style header files. Detailed specification is shown in Table 1.

**Table 1:** Specification of all sensors in sensor unit.

Device type	Model name	Manufacturer	Specification	
Hyperspectral part				
Hyperspectral camera (1 camera per session)	FX10	Specim, Finland	Number of bands	448
			Wavelength range	400–1000 nm
			Spectral resolution	1.34 nm
			Number of pixels	1024
			Signal-to-noise ratio (SNR)	600:1
			Field-of-view (FOV)	38°
	FX17	Specim, Finland	Number of bands	224
			Wavelength range	900–1700 nm
			Spectral resolution	3.57 nm
			Number of pixels	640
			SNR	1000:1
			FOV	38°
Geometry part				
IMU	MW-AHRSv1	NTREX LAB, S. Korea	Static error	< 0.2°
			Dynamic error	< 2°
			Euler angle resolution	0.01°
			Response time	< 1 ms
Stereo camera	ZED	Stereolabs, USA	Depth range	0.5–25 m
			Depth accuracy	< 2% up to 3 m
				< 4% up to 15 m
			Image resolution	1920 × 1080 pixel
			Baseline	120 mm
			FOV	90° (H) × 60° (V) × 100° (D)
LIDAR	RPLidar-A3	Slamtec, China	Distance range	White object: 25 m
				Dark object: 10 m
			Minimum ranging	0.2 m
			Range Resolution	≤ 1% of the range (≤ 12 m)
				≤ 2% of the range (> 12 m)
			Accuracy	1% of the range (≤ 3 m)
				2% of the range (3–5 m)
				2.5% of the range (5–25 m)
			Sample rate	16,000 times/s
			Scan rate	15 Hz
			Angular resolution	0.225°

A halogen lamp was used in light source unit. Halogen gas is injected into a quartz glass tube that seals a tungsten filament and is heated when electricity is applied to the filament to emit incandescent light. It has the characteristic of having a high continuous emission of light in both the visible and infrared regions. It has been used in many hyperspectral sensor studies and in the field because it guarantees stable light throughout the measurement wavelength band of optical sensors^40^. In this system design, a 1000 W, 189 mm J-type linear halogen lamp is mounted within a rectangular illumination reflector. The reflector allows the light source to be directional, and because of the characteristics of the sensor scanning the linear pushbroom, the range of illumination by the linear halogen lamp may sufficiently include the FOV of the sensor. Therefore, it was possible to obtain a homogeneous amount of light within the illumination range. A high-power light source of 1000 W can saturate the 12-bit CCD of the hyperspectral camera; therefore, a sufficient radiometric resolution can be expected.

The geometry part consists of an inertial measuring device (IMU), a stereo camera, and a LIDAR. IMU is the key part to project the hyperspectral pixels. The IMU provides information on the scanning angle of each line of the hyperspectral image. The pixel-wise projection of the image is carried out using the scanning angle information and the FOV value of the hyperspectral camera. The image projection process will be described in detail in the “Data processing” section.

The IMU sensor is MW-AHRSv1 from NTREX LAB, Korea. It measures the sensor unit’s rotation by the driver unit and the movement of the entire system including position errors from uneven mine roads. It is originally consisted of accelerometer and gyroscope, providing linear acceleration and angular rate data. The IMU’s firmware converts the data to three-axis rotation angle (roll, pitch, and yaw) format. Each angle value is used in data processing for projecting the pixels of the hyperspectral image. IMU generally includes drift-induced error value in the data. This is because high-frequency noise is eliminated while low-frequency noise is amplified during the integration process of obtaining angle values from the gyroscope. The MW-AHRSv1 performs drift correction that limits dynamic errors to 2° or less through a Kalman filter built into the firmware. In the case of roll and pitch values, this correction is valid through gravity value, but the yaw value is vulnerable to drift. As a result of measuring the drift of the yaw value in the static laboratory condition, it was confirmed to be about 0.3° per minute. In this study, since it takes about 3–5 min to obtain one dataset, the yaw drift error obtained from one sequence was expected to be 1.5°. The rotation of the sensor unit is collected at 0.5 s intervals.

Stereo camera and LIDAR are auxiliary sensors for visual analyzing and future data processing. RGB pair images were collected using stereo cameras, and a 2D point cloud was collected using LIDAR. Stereo camera is a clustering of two cameras with a certain distance as a baseline. It extracts depth information using the binocular disparity that appears in the difference between the left and right images. 3D depth information is extracted using the pixel position difference of each object in a RGB image pairs by stereo camera’s Python Application Programming Interface (API). LIDAR periodically collects the distances for each rotating direction based on the observation origin via a fast-rotating laser distance meter, providing 2D depth information. The geometry part is mounted right next to the hyperspectral camera to observe in the same direction as the observation direction of the hyperspectral part and acquire geometric information that matches each line of the hyperspectral image. The IMU data for recording the position information of the sensor unit in real time are added to the geometric information. The stereo vision system used in this study was a Stereolabs ZED. The depth detection range is 0.5–25 m, and the resolution is 1920 × 1080 pixels. The LIDAR is RPLIDAR-A3 from Slamtec, and the maximum detection range is 25 m; however, the detection range can be reduced to 10 m for black objects. The LIDAR mirror rotated 360° and acquired up to 16,000 samples per second. Detailed specification of IMU, Stereo camera and LIDAR is shown in Table 1.

The Driver unit is divided into a rotary stage A for rotating the entire sensor unit and a Rotary stage B for rotating the light source (Figs. 6 and 7). Rotary Stage A is Specim RS10, compatible with the Specim FX series hyperspectral cameras. Rotary stage B was defined as Misumi AR -1200. The Sensor unit and light source part are mounted on independent rotary stages to prevent the SNR decrease of the sensor unit owing to the heat of the light source and to disperse the load applied to the motor by distributing the weight. The rotation range was 0°–180°. In both rotary stages, the axis of rotation was parallel to the central axis of the shaft or tunnel to be scanned (coaxial). This design involves scanning all the surfaces with a pushbroom scan of the sensor unit, except for the bottom surface of the target. Depending on the average distance from the exploration origin to the outcrop surface, the height of the driving part can be freely adjusted on an aluminum beam erected perpendicular to the floor.

The main control of the hyperspectral module used a Windows PC with an Intel i7 CPU. Specim's hyperspectral part control program allows scan options (exposure time, frame rate, spectral and spatial binning, etc.), rotation speed, and scanning range of the driver unit to be set. A time server is installed in the PC to share preset time information between each piece of equipment. The hyperspectral images were stored on an SSD on a PC. The geometry part was controlled by the NVIDIA Jetson Nano developer board and used a dedicated Ubuntu Linux OS. Using the Python code developed in this study, RGB images, depth information, and IMU position information of stereo cameras were acquired in Python NumPy array format, and simultaneously, LIDAR point cloud data were collected. Each material was stored on an SSD external storage connected by a USB 3.0. All Controller units were wired through an intranet router built into the system to transmit and receive data. The router supports the wireless Internet (Wi-Fi) and allows control even at a certain distance from the system. This design allows users to operate the system remotely through a laptop or tablet PC, and allows users to work safely at a distance of up to 15 m from the equipment in dangerous areas. In addition, wireless Internet can be used at sites where smart mining environments are established. Hence, it was designed with the possibility that equipment will be remotely controlled in a safe environment, such as outside the mine.

The GNSS signal cannot be received in an underground space. This problem causes not only the barrier to deciding precise location, but also makes it impossible to obtain precise time information during the exploration. Since each device in the system was operated through each controller board or PC, a time synchronization process was required for data processing after acquiring data between each device. The time server provided a standard time within the system so that data acquired from each device was chronologically synchronized and stored the time tag. For example, if the hyperspectral module acquires data at 10 frames (line) per second and the geometric module at 2 Hz, the two modules were synchronized via time server, so that geometric information for each line can be matched through interpolation in time scale. While preparing for exploration on the ground, the time server was initialized and synchronized to precise International Standard Time via mobile internet.

The equipment developed in this study consumes approximately 1000 W of power from a light source, a sensor, and a controller unit of approximately 150 W of power, and a high output power of at least 1200 W is required. However, it is difficult to freely use electricity at mining sites or in civil engineering environments. To utilize external power, power must be installed in a certain area; therefore, mobility is poor or the exploration range is limited. Although electricity can be generated by mobile gasoline generators, it is excluded because of the risk of suffocation of workers owing to exhaust gas when used in underground mines. Considering this field situation, in this study, four DC 12 V 130 Ah lithium iron phosphate battery packs were connected in parallel and then converted into 220 V AC using a power inverter to supply electricity. All the payloads were mounted on a cart-type platform for mobility (Fig. 6). As most target environments in this study had unpaved roads, air-injected rubber tires that are stable for vibration and movement were installed. The cart consisted of two floors with a heavy power supply unit on the lower floor, an aluminum profile column equipped with a rotation/sensor unit, and the controller unit’s intranet router on the upper floor.

### Data acquisition

After installing a tape measure in the 20 m target section of the exploration shaft, a test dataset was acquired (Fig. 2). The average scanning range of the hyperspectral camera on the tunnel surface was approximately 1.72 m. It was calculated based on the average width of the mine tunnel (4–5 m), and the camera’s FOV (38°). On the last lines of the image in each set, 99% and 5% reference panels were installed on the tripod as close to the mine wall as possible. In this test data acquisition, a Specim FX10 VNIR hyperspectral camera was mounted. The frame rate of the hyperspectral camera was set to 10 frames/s, a height of 1 m, a driver unit was set to rotate at 0.1°/s, and a full range of 171.3°. The Ground Control Point (GCP) for georeferencing was triangulated and marked on both walls of the mine at intervals of 5 m, at a height of approximately 1 m from the bottom of the mine. The system was remotely controlled using a wireless router performed at a distance of 3–5 m from the device to minimize the impact of light sources owing to user movement and exploration convenience.

The hyperspectral camera produces a dark image by closing the shutter between 100 frames at the beginning of each scan set. The dark image is used to correct the thermal noise between each pixel of the camera and to measure a basic biased digital pixel value according to the amount of dark current of each pixel in a state where light is blocked. Thereafter, the main image was acquired using the frame rate and rotation range settings. While the Driver unit rotates, the IMU and stereo camera acquire data once every 0.5 s, and the stereo camera generates depth data in real-time stored with position and time information. LIDAR obtained distance information according to time and rotation angle by rotating 15 times per second during the data acquisition of one set.

In addition, 10 samples of iron ore cores at the mine site were collected and imaged in the laboratory for further analysis and interpretation of the acquired hyperspectral images. For hyperspectral imaging of iron ore cores, Specim FX10 hyperspectral camera was mounted on the desktop conveyor belt system, and the light source was set to an environment as similar to the exploration conditions as possible using a halogen lamp. The conveyor belt system facilitates the continuous and spatial scanning of objects by moving samples at a constant speed according to the frame rate of a fixed pushbroom camera. The top of the sample tray was equipped with a Spectralon® 99% diffuse reflectance reference, and cross-track illumination and radiation corrections were performed. Then, a 50 × 50 pixel region of interest (ROI) was set for the center of each sample, and an average reflectance spectrum for the ROI was derived to produce a spectrum library for high-grade iron ore. It showed a gentle rise near a wavelength of 700 nm and a spectrum of depression after a wavelength of 960 nm. In the region of 450 nm or less, the reflectance value is abnormally amplified owing to the noise characteristics of the device itself.

### Data processing

**Figure 8:**
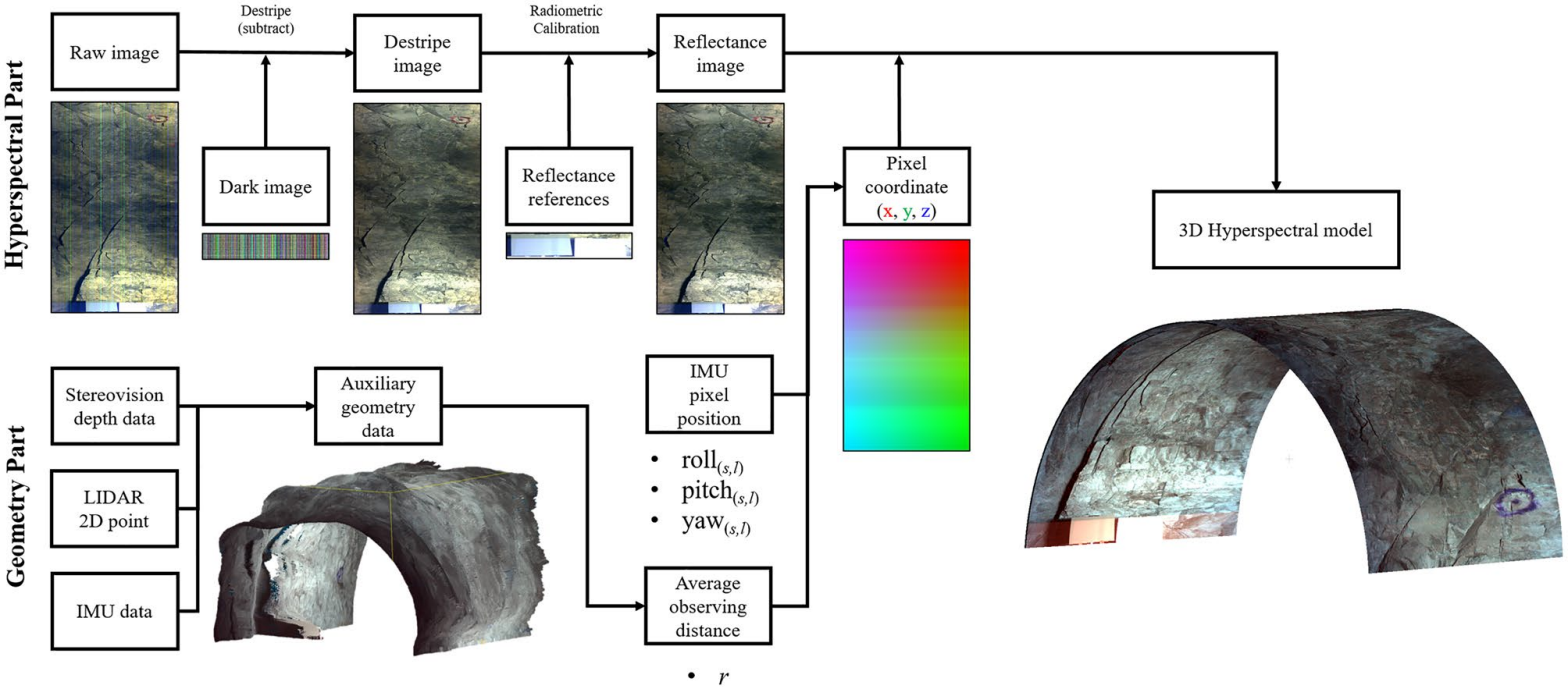
Data processing of hyperspectral data acquired in this study: From image calibration to 3D point projection. Hyperspectral Part pipeline: Raw hyperspectral image is destriped with dark image. The destriped digital number (DN) image is calibrated with reflectance references into the reflectance image. Geometry Part pipeline: Stereo depth data and LIDAR 2D point data are conjugated with IMU data that makes 3D auxiliary geometry data for calculating average observing distance (*r*). The *r* and IMU’s roll, pitch, and yaw data make 3D coordinates of each pixel. Reflectance images or mineral mapping data can be projected into a 3D model by pixel coordinate data.

First, hyperspectral images were calibrated (Fig. 8). Prior to data processing, a preprocessing process was applied to reduce noise and correct various distortions included in the data. Stripe noise is generated because each pixel of the line scanning camera has different light-receiving characteristics, or thermal noise, and leaves linear noise in the sample direction of an image. An intermediate value was calculated for each wavelength band (λ) and sample (*s*) from the dark image acquired at the beginning of each dataset, and the dark current pixel value of each CCD was determined. Thereafter, destripe correction was performed by subtracting the dark current pixel value (*DN*_*dark*_) from the main raw hyperspectral (*DN*_*raw*_) image for each line.

1 $$DN_{destriped} \left( s, \lambda \right) = DN_{raw} \left( s, \lambda \right) - DN_{dark} \left( s, \lambda \right)$$

Next, radiometric calibration was performed using 99% and 5% reflectance reference panels to convert destriped DN (*DN*_*destriped*_) into reflectance values. The ROI was set on pixels corresponding to the panels in the image to obtain an average DN value (*DN*_*reference*_) and then match it to the reflectance value for each wavelength band provided in the reference (*Reflectance*_*reference*_). The DN value of each pixel of the image was linearly interpolated into reflectance using the DN-to-reflectance conversion information of the reference.

2 $$Reflectance \left( {s,l,\lambda } \right) = \frac{{DN_{destripbed} \left( {s,l,\lambda } \right)}}{{DN_{reference} \left( \lambda \right)}}{ } \times Reflectance_{reference} \left( \lambda \right)$$

**Figure 9:**
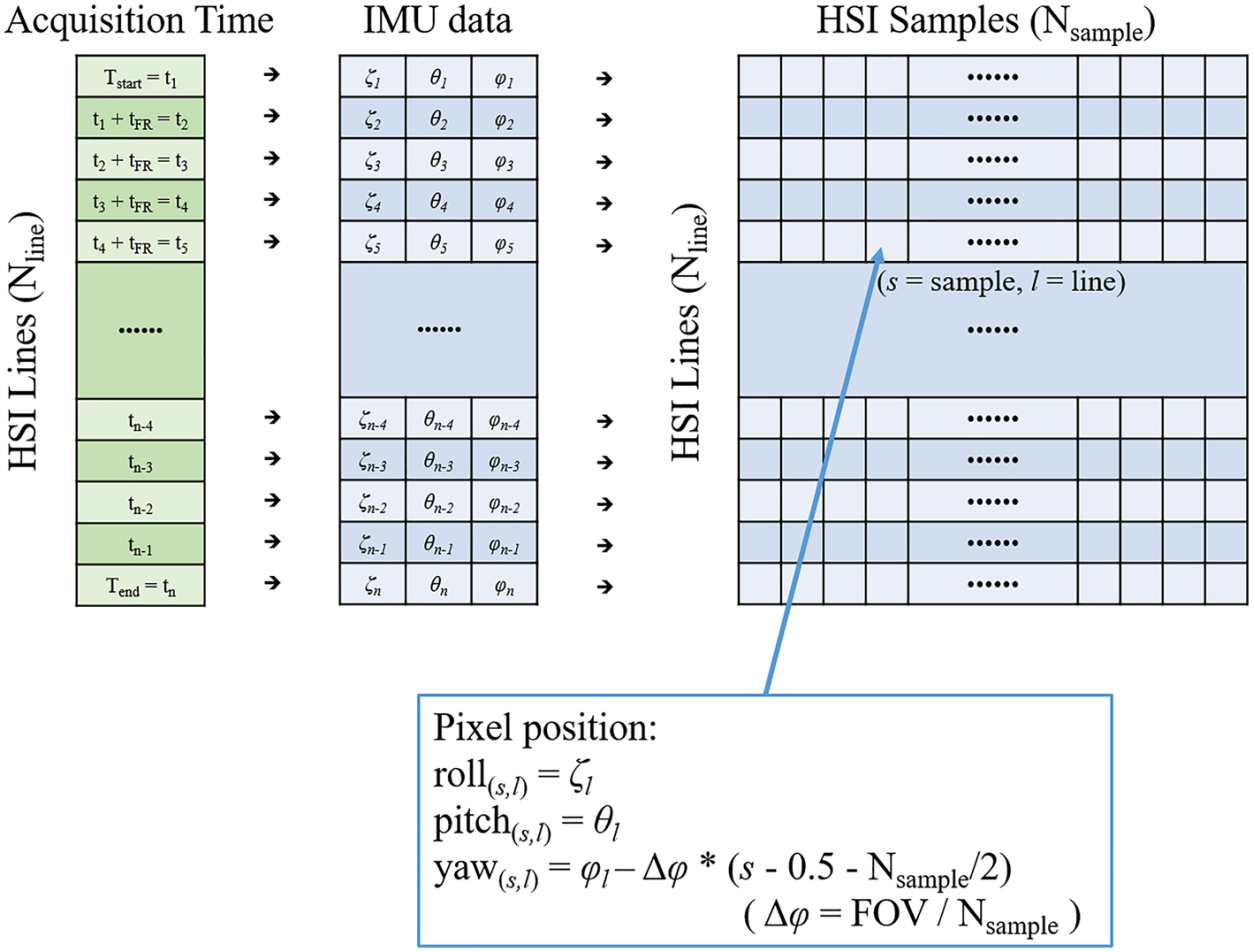
Extracting position information of each pixel in hyperspectral image pixels. Roll, pitch, and yaw for pixel (*s*, *l*) corresponding to an arbitrary line (*l*) and sample (*s*). The pixel position of each HSI pixel can be calculated as the formula in the figure.

Finally, all pixels were projected onto a half-cylindrical tunnel plane using IMU information to create a geometrically tunnel-shaped image (Fig. 9). The dataset has the time information at the data acquisition: Start time (T_start_) and end time (T_end_) of the hyperspectral image collection. Each line of the hyperspectral image was scanned at a certain period (10 frame/s in test) and number of the line (*l*), so time information can be interpolated to the specific time (t_*l*_) at which each line was taken. IMU position data (roll, pitch, and yaw) also has the time tag on each sampling. When IMU data is interpolated for the acquisition time for each line of the image (t_*l*_), posture information at the time when each line is acquired is obtained (roll = *ζ*_*l*_, pitch = *θ*_*l*_, yaw = *φ*_*l*_). Meanwhile, the Specim FX10 hyperspectral camera has 1024-pixel samples (N_sample_) in a line, and the FOV is 38°. So, each pixel had angle size (IFoV: Instantaneous Field of View) of about 0.037° (Δφ). It could be assumed that each pixel (sample) in one line observed the target object while rotating with respect to the center of the camera according to the order in which they are arranged in the yaw direction. Therefore, the yaw value currently being viewed by the sample could be obtained by adding or subtracting the Δφ value(s) from the yaw value of the entire line (*φ*_*l*_) according to each sample number. Therefore, yaw of each pixel at sample number *s*, line number *l* could be calculated as following formula (3).

3 $$yaw \left( {s,l} \right) = \varphi_{l} - \Delta \varphi {*}\left( {s{ }{-}{ }0.5{ }{-}\frac{{{ }N_{sample} }}{2}} \right)$$

The roll and pitch value at sample number *s*, line number *l* is same as the whole line’s position value.

4 $$roll\,\left( {s,l} \right)\,=\,\zeta_{l}$$

5 $$pitch\,\left( {s,l} \right)\,=\,\theta_{l}$$

The drift error of the IMU’s yaw value was predicted to be about 1° per data collection. In each acquisition, the platform of the system was fixed, so only the sensor unit rotated coaxially based on the tunnel. This movement changes the IMU’s pitch value, but there was no significant change in the yaw value during the exploration. Therefore, yaw drift errors were ignored.

**Figure 10:**
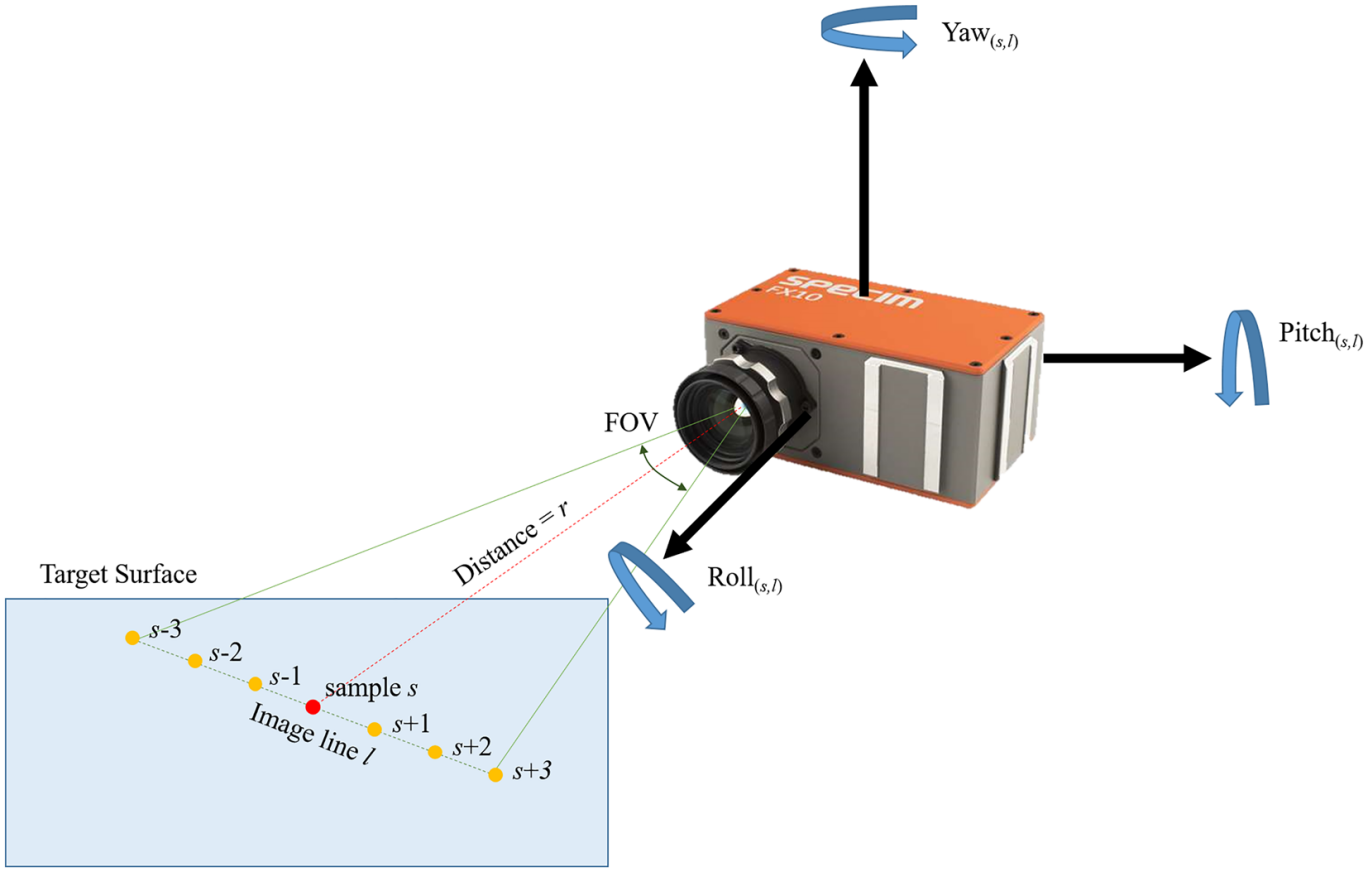
Position information of the hyperspectral camera and each image’s pixel at line number *l*, sample number *s*, sensor to target surface distance *r*.

In this study, mine shafts and tunnels were approximated in a semi-cylindrical shape. Thus, each pixel was projected to have a radius (*r*) equal to the distance from the sensor to the target surface (observing distance) of each pixel in a cylindrical coordinate system, at an angle corresponding to the pixel's roll, pitch, and yaw values. Cylindrical projection process was given by following process. (1) Assumed a spherical coordinate azimuth angle = 0, elevation angle = *θ*_*l*_, distance *r*: Convert the coordinate system from spherical to Cartesian. (2) Added a yaw rotation effect on each pixel at y-coordinate. (3) Rotated the coordinates by the roll angle (*ζ*_*l*_) with Euler Angle Rotation [36]. As a result, Cartesian coordinate information of each pixel was constructed. Hyperspectral pixels could be projected as 3D model via coordinate information (Fig. 10). Meanwhile, accurate observing distance *r* of each pixel could be determined only when geometric information of the mine tunnel is acquired. The auxiliary geometry data; stereo camera depth information and LIDAR data could make the 3D model of the mine. However, fusing the auxiliary geometry data and hyperspectral point cloud is under development. In this study, observing distance *r* was given as 2.1 m; average of the origin of the sensor to observing surface distance by following auxiliary geometry data.

Auxiliary point cloud data was constructed by stereo camera, LIDAR, and IMU. Generally, LIDAR data is more powerful than stereo images for constructing accurate point clouds^41–43^. Yet, stereo vision meets the goal of this study to analyze the continuous distribution of materials. Stereo vision data provides RGB images and includes RGB color information even after the point cloud is constructed, so it can be directly compared and analyzed with hyperspectral images. However, monochromatic LIDAR provides only the geometric information and is difficult to analyze the color and visible pattern of objects. Thus, a stereo camera was used as main geometric sensor, and a LIDAR was used as an auxiliary sensor. The stereo camera’s firmware natively provides the depth and orthogonal coordinate of each image’s pixel. By using stereo images’ 3-axis coordinates, 10 horizontal lines in the center are collected from each stereo image data every 0.5 s during the hyperspectral dataset is under acquisition. Each stereo image line set is rotated by IMU rotation angle information of the Sensor Unit, and all of them are stacked as auxiliary geometry data. If the difference in the geometry data of stereo images and the LIDAR point cloud for a specific location are more than a certain threshold, they are set as outliers, and the point is removed from the stereo image geometry of the location and then interpolated to correct the error. This outlier occurs because objects with abnormal reflectance (such as shiny metal pipes) in the mine cause interference when acquiring the geometric data. All data processing procedures are summarized in Fig. 8.

## Data Availability

The datasets of this study are available from the corresponding author on reasonable request.
